# Non-urate transporter 1, non-glucose transporter member 9-related renal hypouricemia and acute renal failure accompanied by hyperbilirubinemia after anaerobic exercise: a case report

**DOI:** 10.1186/s12882-019-1618-1

**Published:** 2019-11-26

**Authors:** Yoshitaka Furuto, Mariko Kawamura, Akio Namikawa, Hiroko Takahashi, Yuko Shibuya, Takayasu Mori, Eisei Sohara

**Affiliations:** 1 Department of Hypertension and Nephrology, NTT Medical Centre, Tokyo, 5-9-22, Higasi-Gotanda, Shinagawa-ku, Tokyo, 141-8625 Japan; 2 Department of Nephrology, Tokyo Medical and Dental University, 1-5-45, Yushima, Bunkyo-ku, Tokyo, 113-8519 Japan

**Keywords:** Renal hypouricemia, Acute renal failure with severe loin pain and patchy renal ischemia after anaerobic exercise, Hyperbilirubinemia, Acute kidney injury

## Abstract

**Background:**

Renal hypouricemia (RHUC) is an inherited heterogenous disorder caused by faulty urate reabsorption transporters in the renal proximal tubular cells. Anaerobic exercise may induce acute kidney injury in individuals with RHUC that is not caused by exertional rhabdomyolysis; it is called acute renal failure with severe loin pain and patchy renal ischemia after anaerobic exercise (ALPE).

RHUC is the most important risk factor for ALPE. However, the mechanism of onset of ALPE in patients with RHUC has not been elucidated. The currently known genes responsible for RHUC are *SLC22A12* and *SLC2A9*.

**Case presentation:**

A 37-year-old man presented with loin pain after exercising. Despite having a healthy constitution from birth, biochemical examination revealed hypouricemia, with a uric acid (UA) level of < 1 mg/dL consistently at every health check. We detected acute kidney injury, with a creatinine (Cr) level of 4.1 mg/dL, and elevated bilirubin; hence, the patient was hospitalized. Computed tomography revealed no renal calculi, but bilateral renal swelling was noted. Magnetic resonance imaging detected cuneiform lesions, indicating bilateral renal ischemia. Fractional excretion values of sodium and UA were 0.61 and 50.5%, respectively. Urinary microscopy showed lack of tubular injury. The patient’s older sister had hypouricemia. The patient was diagnosed with ALPE. Treatment with bed rest, fluid replacement, and nutrition therapy improved renal function and bilirubin levels, and the patient was discharged on day 5. Approximately 1 month after onset of ALPE, his Cr, UA, and TB levels were 0.98, 0.8, and 0.9 mg/dL, respectively. We suspected familial RHUC due to the hypouricemia and family history and performed genetic testing but did not find the typical genes responsible for RHUC. A full genetic analysis was opposed by the family.

**Conclusions:**

To the best of our knowledge, this is the first report of ALPE with hyperbilirubinemia. Bilirubin levels may become elevated as a result of heme oxygenase-1 activation, occurring in exercise-induced acute kidney injury in patients with RHUC; this phenomenon suggests renal ischemia-reperfusion injury. A new causative gene coding for a urate transporter may exist, and its identification would be useful to clarify the urate transport mechanism.

## Background

Renal hypouricemia (RHUC) is a common inherited heterogenous disorder, caused by faulty urate reabsorption transporters in the renal proximal tubular cells. Furthermore, RHUC does not originate from congenital purine metabolism abnormalities or secondary hypouricemia [1, 2]. After anaerobic exercise, such as sprinting, an acute kidney injury not related to myoglobin may develop. This acute kidney injury is called acute renal failure with severe loin pain and patchy renal ischemia after anaerobic exercise (ALPE), and it is not caused by exertional rhabdomyolysis.

ALPE is also known as exercise-induced acute kidney injury (EIAKI) and is one of the complications of RHUC [1]. Moreover, RHUC is an important risk factor for ALPE. The mechanism of ALPE with RHUC is not sufficiently known, but various hypotheses include active oxygen scavenging insufficiency/renal ischemia and acute uric acid nephropathy. RHUC is a condition that reduces reabsorption in the proximal tubule or promotes secretion, resulting in accelerated uric acid (UA) clearance in the kidneys and markedly decreases serum UA levels. Genetic mutations have been reported to play a role in RHUC; most cases of RHUC are associated with mutation of the genes *SLC22A12* [encoding urate transporter 1 (URAT1)] [3] and *SLC2A9* [encoding glucose transporter member 9 (GLUT9)] [4–9], but there are exceptions as well. We report the case of a patient with ALPE with hyperbilirubinemia and RHUC who did not have the characteristic genetic mutations.

## Case presentation

A 37-year-old man presented with the chief complaint of loin pain. He had no abnormalities in growth and development and had a healthy constitution; however, he once had an approximately 1-week episode of convalescence for loin pain after exercising in junior high/high school. Hypouricemia was noted on every biochemical examination in health check with a UA level of < 1 mg/dL. However, until now, his renal function and urine findings had been normal, and there was no history of examination at a medical institution. Bilateral loin pain developed during walking. The patient expected an improvement in the pain from walking; hence, he engaged in intensive exercise, including swimming for 1 h, fast walking / jogging for 2 km, and active muscle training for approximately 2 h. However, the pain worsened and persisted. He was then examined at the Department of Urology at our hospital. Renal calculi were ruled out via computed tomography (CT), but with a creatinine (Cr) level of 3.7 mg/dL, acute kidney injury with hyperbilirubinemia was diagnosed. The patient was then examined at the Department of Nephrology at our hospital. The following day, renal function further worsened with a Cr level of 4.1 mg/dL, and the patient was hospitalized on an emergency basis for detailed testing and treatment.

He had no notable medical history and no constitutional jaundice. The patient’s older sister was reported to have hypouricemia, but the UA level was unknown. He also had no history of allergy and was a non-smoker and an occasional drinker. His physical characteristics were as follows: height, 166 cm; weight, 66.8 kg; body mass index, 24.2 kg/m^2^; blood pressure, 135/53 mmHg; heart rate, 53 beats/min; body temperature, 37.0 °C; lucid; no anemia of the palpebral conjunctiva; no jaundice of the bulbar conjunctiva; no oral cavity findings; no swelling of the cervical lymph nodes; bilateral costovertebral angel tenderness; no pedal edema; no arthralgia; no cutaneous findings; and no neurological findings.

The blood and urine test results of the patient are shown in Table 1. The blood test results revealed renal function damage, hyperbilirubinemia in indirect bilirubin dominance, slight increase in C-reactive protein level, elevated fibrinogen levels, slight increases in creatine kinase (CK) and myoglobin levels, and hypouricemia. However, no data could be found suggestive of a collagen disease or vascular disease, and there were no findings indicative of hemolysis without anemia and elevated LDH.

**Table 1:** Laboratory data at admission (Day 2)

Urinalysis		Biochemistry/Immunological test/Coagulation test			
Protein (−)	±	TP (6.4~8.1 g/dL)	7.1 g/dL	HbA1c (4.6~6.2%)	5.2%
Occult blood (−)	–	Alb (3.9~4.9 g/dL)	4.0 g/dL	TSH (0.35~4.94 μIU/mL)	1.447 μIU/mL
Glucose (−)	–	UA (3.7~7.0 mg/dL)	2.7 mg/dL	FT4 (0.7~1.48 ng/dL)	0.83 ng/dL
Bilirubin (−)	–	BUN (7.2~20.0 mg/dL)	38.1 mg/dL	IgG (870~1700 mg/dL)	1433 mg/dL
Red blood cell (< 1/HPF)	< 1/HPF	Cr (0.5~1.1 mg/dL)	4.14 mg/dL	IgA (110~410 mg/dL)	163 mg/dL
Protein content (< 150 mg/gCr)	98 mg/gCr	eGFR (≧60 mL/min/1.73m^2^)	15 mL/min/1.73 m^2^	IgM (33~190 mg/dL)	129 mg/dL
PH (4.8–7.5)	5.5	TB (0.2~1.0 mg/dL)	2.5 mg/dL	C3 (86~160 mg/dL)	103 mg/dL
Myoglobin (≦10 ng/mL)	12.1 ng/mL	DB (0~0.4 mg/dL)	0.6 mg/dL	C4 (17~45 md/dL)	24.5 mg/dL
α1MG (≦8.0 mg/L)	11.91 mg/L	AST (10~40 IU/L)	23 IU/L	CH50 (30~45 U/mL)	49 U/mL
NAG (≦11.5 U/L)	3.8 U/L	ALT (5~45 IU/L)	21 IU/L	Antinuclear Ab (≦40)	1:40
β2MG (≦250 μg/L)	415 μg/L	ALP (104~338 IU/L)	149 IU/L	Anti-dsDNA-Ab (−)	(−)
FENA (< 1.0%)	0.61%	γ-GT (16~73 IU/L)	24 IU/L	Anti-Sm-Ab (−)	(−)
FEUA (5.5~11.1%)	50.5%	LDH (120~245 IU/L)	218 IU/L	Anti-MPO-ANCA (−)	(−)
Complete blood cell count		CK (50–230 IU/L)	300 IU/L		
White blood cell (3100~9500/μL)	9100/μL	Na (136~145 mEq/L)	138 mEq/L	Anti-PR3-ANCA (−)	(−)
Neutrophil (43.7~76.4%)	76.0%	K (3.6~4.8 mEq/L)	4.5 mEq/L	Serum-Myoglobin (≦60.0 μg/mL)	67.1 ng/mL
Lymphocyte (16.2~47.6%)	14.5%	Cl (99~109 mEq/L)	102 mEq/L	Lactate (4.0~16.0 mg/dL)	8.9 mg/dL
Monocyte (2.9~7.9%)	8.5%	cCa (8.4~10.4 mg/dL)	9.2 mg/dL	PT (70~130%)	110%
Eosinophil (0.6~9.0%)	0.8%	IP (2.2~4.6 mg/dL)	3.2 mg/dL	PT-INR (1.0 ± 0.15)	0.96
Red blood cell (401~540 × 10^4^/μL)	445 × 10^4^/μL	Glu (70~109 mg/dL)	88 mg/dL	APTT (24.0~38.0 s)	25.1 s
Hemoglobin (13.5–16.9 g/dL)	13.8 g/dL	TC (130~219 mg/dL)	193 mg/dL	Fibrinogen (170~360 mg/dL)	389 mg/dL
Hematocrit (39.0~51.2%)	40.2%	TG (30~149 mg/dL)	69 mg/dL	FDP (< 10.0 μg/mL)	2.1 μg/mL
Platelet (15.1~34.9 × 10^4^/μL)	21.1 × 10^4^/μL	CRP (≦0.3 mg/dL)	1.6 mg/dL	D-dimer (< 1.0 μg/mL)	0.6 μg/mL

The reference values for each variable are presented in parentheses to the right or under it

*α1MG* α1-microglobulin, *NAG* N-Acetyl-β-D-glucosaminidase, *β2MG* β2-microglobulin, *FENA* Fractional excretion of sodium, *FEUA* Fractional excretion of uric acid, *TP* Total protein, *Alb* Albumin, *UA* Uric acid, *BUN* Blood urea nitrogen, *Cr* Creatinine, *eGFR* Estimated glomerular filtration rate, *TB* Total bilirubin, *DB* Direct bilirubin, *AST* Aspartic aminotransferase, *ALT* Alanine aminotransferase, *ALP* Alkaline phosphatase, *γ-GT* γ-glutamyl transferase, *LDH* Lactate dehydrogenase, *CK* Creatine kinase, *Na* Sodium, *K* Potassium, *Cl* Chlorine, *cCa* Corrected calcium, *IP* Inorganic phosphorus, *Glu* Glucose, *TC* Total cholesterol, *TG* Total glyceride, *CRP* C-reactive protein, *HbA1c* Hemoglobin A1c, *TSH* Thyroid stimulating hormone, *FT4* Free thyroxine, *IgG* Immunoglobulin G, *IgA* Immunoglobulin A, *IgM* Immunoglobulin M, *Ab* Antibody, *dsDNA* Double- stranded DNA, *Sm* Smith, *MPO-ANCA* Myeloperoxidase-anti-neutrophil cytoplasmic antibody, *PR3-ANCA* Proteinase-3-anti-neutrophil cytoplasmic antibody, *PT* Prothrombin time, *PT-INR* Prothrombin time-international normalized ratio, *APTT* Activated partial thromboplastin time, *FDP* Fibrinogen degradation products

**Fig. 1:**
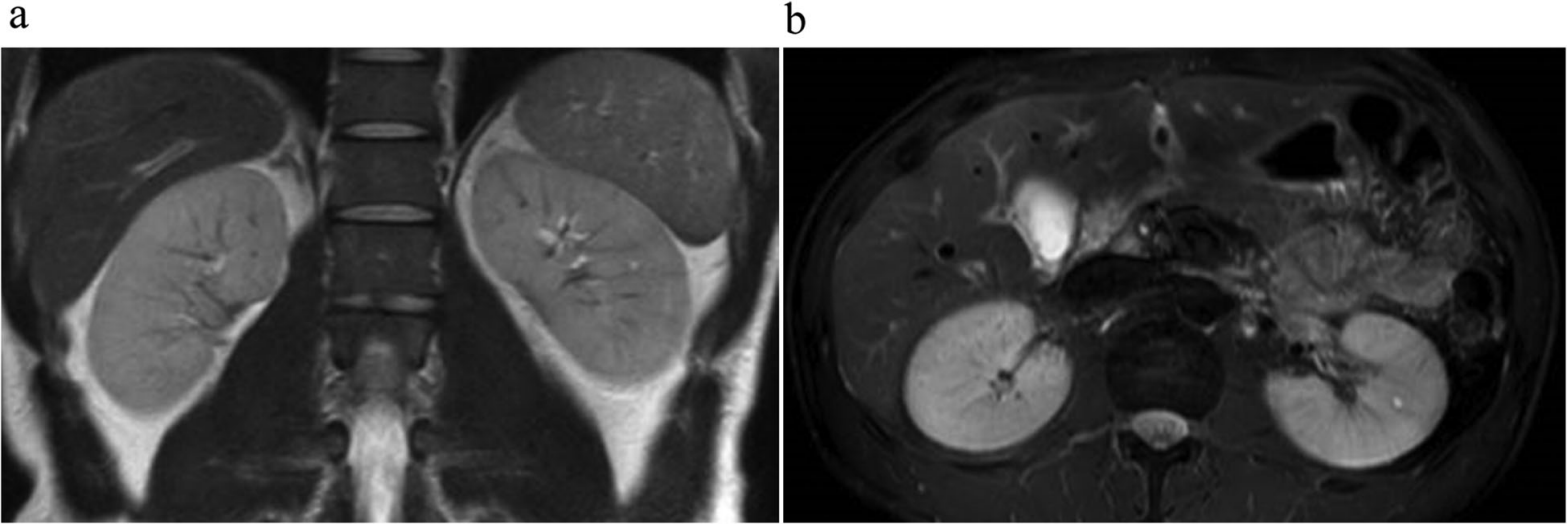
Renal MRI image. Cuneiform low-signal region found in the T2-weighted renal MRI image, indicating heterogeneous ischemia. **a** Coronal, **b** Axial. MRI, magnetic resonance imaging

The urine test results did not reveal any abnormal urinary findings. There were no urinary microscopic evidence, tubular epithelial cells, granular cast, epithelial cell cast, uric acid crystals, microscopic hematuria, and eosinophiluria. However, there were aciduria and mild elevation in myoglobin, whereas tubular injury markers were almost normal. Prerenal failure was suggested by a fractional excretion of sodium (FENA) level of 0.61, and fractional excretion of UA (FEUA) was markedly elevated at a level of 50.5%. CT examination revealed bilateral renal swelling, but there were no hepatobiliary structural abnormalities and no other abnormal findings. Contrast-enhanced CT was not performed because of the renal dysfunction. Instead, renal magnetic resonance imaging (MRI) was performed, and the T2-weighted image showed a cuneiform low-signal area suggestive of heterogeneous ischemia (Fig. 1a, b).

For the differential diagnosis, medical history was gathered, and laboratory investigations were conducted. There had been no issues during growth and development; the patient had never experienced any liver injury, and bilirubin levels had been within the normal range at every yearly health check. There was no past history of fatty liver, viral hepatitis, or congenital liver disease. Furthermore, hepatitis B virus surface antigen, hepatitis B virus core antibody, and hepatitis C virus antibody are all negative. No chronic liver injury, neurological injury, or urinary abnormalities were detected. Based on these findings, Fanconi syndrome and Wilson’s disease were excluded. No inappropriate secretion of antidiuretic hormone, malignant tumors, diabetes, or diarrhea was detected. The patient did not receive NSAIDs, antihyperuricemics, herbal supplements, nephrotoxic drugs, or contrast agents. There were no hypotension, infection, or sepsis. Urinary microscopic evidence showed a lack of tubular injury, and tubular injury markers were almost normal; these findings were negative for ATN. Low FENA suggested prerenal acute failure. However, the patient blood pressure was normotensive, and the oral mucous membrane and skin turgor were normal. There was no body weight loss compared to his usual weight, and dehydration was absent. Moreover, the state of the patient fulfilled the diagnostic criteria for ALPE [10]. Based on the findings of renal failure and loin pain after exercise, minor elevation in CK and myoglobin, hypouricemia, and increase in FEUA as well as the MRI findings of bilateral renal swelling and cuneiform low-signal areas, we made a diagnosis of RHUC and ALPE. At that time, the cause of hyperbilirubinemia was unknown.

The patient’s progress was favorable, and he was kept under observation while undergoing bed rest, fluid replacement, and nutrition therapy. On day 4, the Cr level had improved to 3.4 mg/dL, and the patient was discharged on day 5. One month later, his renal function had recovered, with a Cr level of 0.98 mg/dL, and bilirubin levels also normalized to 0.9 mg/dL. However, the UA level was 0.8 mg/dL, indicating severe RHUC. This progress is shown in Table 2. Considering the patient’s familial history of RHUC, we performed genetic testing using next-generation sequencing at the Tokyo Medical and Dental University. We performed comprehensive genetic analysis, including that for *SLC22A12* encoding URAT1 and *SLC2A9* encoding GLUT9, which are genes causing RHUC, and *SLC34A1*, *EHHADH*, *HNF4A*, and *SLC2A2*, which are genes causing Fanconi syndrome; however, the results could not identify the responsible mutation. Based on the patient’s family history, we considered the possibility of a new genetic mutation causing RHUC and considered exome sequencing (full genetic analysis). However, the patient’s family did not provide consent for genetic testing, and thus we were not able to identify the gene in question. To prevent ALPE, we advised the patient to avoid intense exercise; since then, ALPE has not recurred.

**Table 2:** Laboratory data from Day 1 to Day 156

Parameters	Day 1	Day 2	Day 4	Day 8	Day 44	Day 156
UA (3.7~7.0 mg/dL)	3.0	2.7	2.1	2.2	0.8	0.6
Cr (0.5~1.1 mg/dL)	3.66	4.14	3.36	1.56	0.98	0.84
eGFR (≧60 mL/min/1.73 m^2^)	17	15	18	42	70	83
TB (0.2~1.0 mg/dL)	2.4	2.5	1.7	0.9	0.9	0.9
CRP (≦0.3 mg/dL)		1.6	0.5	0.3		0.3
Proteinuria (< 150 mg/gCr)	164	98	116	40	35	33
Urine Occult Blood (−)	(−)	(−)	(−)	(−)	(−)	(−)
Urinalysis Bilirubin (−)	(−)	(−)	(−)	(−)	(−)	(−)
Urinalysis pH (4.8~7.5)	5.0	5.5	6.0	7.0	6.0	7.5
FENA (< 1.0%)	0.33	0.45	0.61	0.71		
FEUA (5.5~11.1%)	48.32	50.5	51.95	47.91	46.41	58.33

The reference values for each variable are presented in parentheses to the right or under it

*UA* Uric acid, *Cr* Creatinine, *eGFR* Estimated glomerular filtration rate, *TB* Total bilirubin, *CRP* C-reactive protein, *FEUA* Fractional excretion of uric acid

## Discussion and conclusions

We report on a case of ALPE accompanied by hyperbilirubinemia with familial RHUC unrelated to mutations in *SLC22A12* encoding URAT1 and *SLC2A9* encoding GLUT9. The clinical course of this case was characterized by recovery from renal failure with improvement of the hyperbilirubinemia. In addition, although we suspected a conventionally known, typical genetic mutation, we did not find such a mutation in next-generation sequencing [11], suggesting familial RHUC via a new genetic mutation that has not previously been identified. According to the clinical practice guidelines for renal hypouricemia (1st edition) [1], the diagnostic guidelines for RHUC require that (1) and (2) listed below be continuously found and (3) be satisfied.

1. Hypouricemia with a serum UA level < 2.0 mg/dL. However, mild RHUC may be present even with a serum UA level of 2.1–3.0 mg/dL [12, 13].
2. Elevated FEUA or UA clearance (CUA). The normal values for FEUA and CUA are 8.3% (range, 5.5–11.1%) and 11.0 (range, 7.3–14.7) mL/min, respectively.
3. Other causes of hypouricemia are ruled out. As other reference items, identifying a genetic mutation responsible for RHUC, having a history of ALPE, and finding a family history of RHUC are satisfied.

The prevalence of RHUC in Japan is reportedly 0.21% in men and 0.39% in women [14], whereas its overall prevalence in Korea is 0.53% [15]; however, the prevalence of hypouricemia in other geographical areas during the same time period has not been extensively studied [16]. The inheritance pattern of RHUC is considered to be autosomal recessive [17]. Its complications include ALPE and urinary calculi. Although the prevalence of ALPE has not been completely elucidated, ALPE accompanied by RHUC occurs approximately 50 times more frequently than ALPE without RHUC [10]; furthermore, 51% of ALPE cases involve patients with RHUC [18], and 6.5–10% of RHUC patients have a history of ALPE [10, 18–20]. Hypotheses for why RHUC patients are prone to ALPE include active oxygen scavenging insufficiency/renal ischemia and acute UA nephropathy. The hypothesis regarding active oxygen scavenging insufficiency/renal ischemia is that because RHUC patients have low levels of UA, which performs scavenging of active oxygen, the increase in active oxygen due to exercise induces renovascular hypertension, and the sustained reduction in renal blood flow causes renal tubule necrosis, leading to ALPE [21–23]. Renal histological findings revealed a high level of renal tubule necrosis [24]. In the hypothesis regarding acute UA nephropathy, exercise by RHUC patients with high FEUA increases UA production, which causes further crystallization of UA in the renal tubules, closing them, and leading to ALPE. However, there are few reports on the pathological findings of UA nephropathy [10, 25–27]. In our patient, since we did not find uric acid crystals or tubular injury in urine microscopy supporting the former hypothesis, we think that ALPE in RHUC may not be due to uric acid direct injury and tubular obstruction. Prerenal renal failure in this case was suggested by < 1% fractional excretion of Na, and a T2-weighted image in MRI showed a cuneiform low-signal area suggestive of heterogeneous ischemia, which supports the hypothesis that renal ischemia may be an onset factor for ALPE. Although the imaging findings were non-specific, a T2-weighted image in MRI demonstrated a cuneiform low-signal area suggestive of heterogeneous ischemia. We hypothesize that this finding may relate to renal vasoconstriction.

There are reports of similar findings [10, 28, 29], a report suggesting renal angina [10], and another indicating reversible renal vasoconstriction [30]. They support the active oxygen scavenging insufficiency/renal ischemia hypothesis.

In imaging tests of ALPE, contrast-enhanced CT similarly resulted in cuneiform residual contrast; however, the use of contrast-enhanced CT requires caution, when there is an acute kidney injury.

Furthermore, during ALPE, we found hyperbilirubinemia in indirect bilirubin dominance without structural abnormalities, but bilirubin levels normalized together with the improvement in renal function. The patient had no prior history of constitutional jaundice, and no findings suggestive of hemolysis. Bilirubin has been suggested to protect cells from oxidative stress induced by reactive oxygen species and free radicals [31]. For example, the physiological significance of neonatal jaundice is believed to be the antioxidant effect of bilirubin that prevents the increased active oxygen from the dramatic increase in the partial pressure of oxygen after birth to reduce tissue damage [32].

The cause of hyperbilirubinemia accompanied by ALPE is not clear in this case. However, from past evidence of a protective mechanism in reactive oxygen species (ROS) generation, we suspect that hyperbilirubinemia in this case may have a relationship with ischemia-reperfusion of the kidney. The evidence on the association between hyperbilirubinemia and ROS is as follows. Heme oxygenase-1 (HO-1) is induced as an adaptive and protective response to tissue injury by ROS. HO-1 degrades heme into carbon monoxide (CO) and biliverdin; the latter is converted to bilirubin [33, 34]. Bilirubin is a potent antioxidant substance [35, 36], which removes ROS produced by inflammation, lipopolysaccharide (LPS), and ischemia-reperfusion damage, prevents tissue injury [37–39], and it has a protective effect on the kidneys [40–42].The benefits of elevated serum bilirubin levels for the prognosis in kidney disease have been reported in several clinical studies [43–47]. Moreover, moderate hyperbilirubinemia improves renal hemodynamics [34, 42, 48].

In renal ischemia-reperfusion injury, production of HO-1 is activated, which promotes bilirubin synthesis [49–52]. This phenomenon is also important to protect kidneys from ROS induction caused by a renal ischemia-reperfusion injury in rats [53–55]. However, in humans, ischemic kidney and acute kidney injury do not usually cause hyperbilirubinemia.

Although acute kidney injury results in the formation of large quantities of active oxygen [56], in RHUC, active oxygen is not sufficiently scavenged [22–24]. HO-1 expression has been found in the renal tubule in ALPE with RHUC, suggesting increase of ROS and ischemic kidney injury [57]. In infants with RHUC, relative antioxidant inadequacy is observed for exercise load in comparison with the control group [23]. Uric acid is a potent antioxidant substance [35], and RHUC may cause antioxidant inadequacy on exercise. The relations of uric acid and bilirubin are not clear, but a negative correlation has been recently demonstrated in the relation between serum bilirubin and serum uric acid levels in patients with ulcerative colitis [58]. From the above, an association may exist between bilirubin and ALPE with RHUC.

Based on these reports, it may be possible that when acute kidney injury occurs under the specific circumstances of active oxygen scavenging insufficiency due to RHUC, an even greater amount of active oxygen is generated, provoking elevated bilirubin as a bioprotective response to the stress induced by renal ischemia-reperfusion injury. In fact, the hyperbilirubinemia in this case was accounted for almost entirely by indirect bilirubin. Moreover, urinary bilirubin never increased, further supporting the significance of indirect bilirubin, which is not water-soluble, throughout the course of the disorder. The elevation of indirect bilirubin, a precursor to bilirubin diglucuronide, implies an activated state of HO-1 to produce bilirubin from heme. Therefore, we hypothesize that elevation of indirect bilirubin may be induced by HO-1, supporting the described hypothesis. Although we did not evaluate it, urinary biopyrrin is a marker of ROS [59], and its evaluation in ALPE and/or RHUC would be more significant to prove the hypothesis.

We presume that in RHUC bilirubin instead of uric acid may increase upon exercise, based on the evidence mentioned above [23, 57, 58]; however, clear evidence is absent.

Our case may be the first report that discusses ALPE as a complication of hyperbilirubinemia, and inferences about this phenomenon suggest a renal ischemia-reperfusion injury, supporting an active oxygen scavenging insufficiency/renal ischemia hypothesis. In future, studies must be conducted that use the accumulation of cases of ALPE with RHUC, in which bilirubin dynamics are of note, and studying this specific pathology will lead to a clarification of the causes of ALPE.

The prognosis for ALPE is favorable, and it resolves with common treatments for acute kidney injury. There are cases where dialysis was performed on a temporary basis, but there are probably no reports of cases requiring maintenance dialysis. In addition, a case of posterior reversible encephalopathy syndrome (PRES) occurring with ALPE has been reported [60, 61]. The pathology of ALPE with RHUC is unknown, and there is no consensus on its prevention or management, but it has been reported that allopurinol reduces urinary urate excretion during exercise in RHUC patients and suppresses UA crystal formation in renal tubule lumens, successfully preventing ALPE. Thus, the xanthine oxidase reductase promoters of allopurinol, febuxostat, or topiroxostat are recommended for use [25, 62]. Most cases of RHUC complicated with ALPE have a usual serum UA level of < 1.0 mg/dL, with almost no cases exceeding 2.0 mg/dL [12, 13, 20, 63–65], suggesting that a serum UA level below a certain value is a risk factor for ALPE. ALPE recurs frequently; thus, guidance on exercise intensity is needed [24], but it is not clear what degree of exercising can be tolerated by RHUC patients. The reason RHUC is prone to complications by urinary calculi is believed to be an increase in urinary urate excretion, and it is found in 7–10% of RHUC cases [63]. RHUC complicated by aciduria has also been reported [66]. Furthermore, RHUC reduces vascular endothelial function and so can be a risk factor for arteriosclerosis [67]. It is associated with a decrease in the amount of glomerular filtration, suggesting that RHUC could be related to renal failure [17, 68].

A relationship has been found between RHUC and the genes *SLC22A12* (encoding URAT1) [3] and *SLC2A9* (encoding GLUT9) [4–9]. URAT1 and GLUT9 are transporters acting in UA reuptake in the proximal tubule. Currently, RHUC caused by a URAT1 genetic mutation is called type 1, and this mutation accounts for 80–90% of RHUC cases in Japanese individuals [63]. RHUC caused by a *GLUT9* genetic mutation is classified as type 2. A relatively recent report has identified GLUT9 as a causative factor for RHUC. The frequency of type 2 is low worldwide; particularly in Japan [6].

In type 1, the UA level is approximately 1.0 mg/dL, and FEUA is 40–90%. In type 2, the UA level is < 1.0 mg/dL and close to 0, whereas FEUA is elevated to 100–150%, suggesting that the effect of UA reuptake by GLUT9 could be greater than that by URAT1 [5–7]. However, cases of RHUC not belonging to either type 1 or type 2 have also been reported [4, 20, 63, 69–71], and our case was also a rare one, falling under this type. Our patient had a UA level of < 1 mg/dL and FEUA of around 50%, which was similar to type 1, but we did not find a URAT1 mutation.

Limitations of the study are that a renal biopsy to confirm renal tubule necrosis as characteristics of ALPE and head MRI to confirm PRES as complication of ALPE were not performed on our patient.

There may still be new causative genes that code for UA transporters. Identifying these genes is important, because they will be useful in clarifying the mechanism of UA transport. To elucidate the molecular mechanism of familial RHUC without mutations in *SLC22A12* encoding URAT1 or *SLC2A9* encoding GLUT9 and to develop methods of prevention and treatment based on the findings, it will be necessary to accumulate more cases and perform genetic analysis in the future.

## Data Availability

Not applicable.

## Abbreviations

ALPE: Acute renal failure with severe loin pain and patchy renal ischemia after anaerobic exercise
CK: Creatine kinase
Cr: Creatinine
CRP: C-reactive protein
CT: Computed tomography
CUA: Uric acid clearance
FENA: Fractional excretion of sodium
FEUA: Fractional excretion of Uric acid
GLUT9: Glucose transporter member 9
LDH: Lactate dehydrogenase
MRI: Magnetic resonance imaging
PRES: Posterior reversible encephalopathy syndrome
RHUC: Renal hypouricemia
TB: Total bilirubin
UA: Uric acid
URAT1: Urate transporter 1
